# The correlation analysis between the appearance anxiety and personality traits of the medical staff on nasal and facial pressure ulcers during the novel coronavirus disease 2019 outbreak

**DOI:** 10.1002/nop2.613

**Published:** 2020-09-13

**Authors:** Xiaodi Kong, Yong Cao, Xiaonian Luo, Lianxiang He

**Affiliations:** ^1^ Department of Urolithiasis Xiangya Hospital of Central South University Changsha China; ^2^ Department of Spine Surgery Xiangya Hospital of Central South University Changsha China; ^3^ Department of Nursing Xiangya Hospital of Central South University Changsha China

**Keywords:** appearance anxiety, COVID‐19, EPQ‐RSC, medical staff, pressure ulcers

## Abstract

**Aim:**

To investigate the psychological status of medical staff with medical device‐related nasal and facial pressure ulcers (MDR PUs) during the outbreak of COVID‐19, analyse the correlation between their psychological status and personality traits, so as to provide a reference for personalized psychological support.

**Design:**

A total of 207 medical staff who were treating the COVID‐19 epidemic from Hunan and Hubei provinces were enrolled in this analytic questionnaire‐based study.

**Methods:**

We used these measures: Eysenck Personality Questionnaire Short Scale (EPQ‐RSC), Social Appearance Anxiety Scale (SAAS), Positive and Negative Affect Scale (PANAS) and demographic information forms online.

**Results:**

Medical staff wearing protective equipment are particularly susceptible to nasal and facial MDR PUs, which is increasing their social appearance anxiety; neuroticism is significantly related to social appearance anxiety and negative emotion. We should pay more attention to their psychological state, cultivate good personality characteristics and reduce negative emotions, and thereby alleviate their MDR PUs‐related appearance anxiety.

## 1 INTRODUCTION

Outbreak of novel coronavirus disease 2019 (COVID‐19) has spread quickly worldwide. At present, nearly 114 nations have reported the confirmed cases. COVID‐19 pandemic is highly infectious, and the threat to global health remains rife (Cheng‐long Xiong & Qing‐wu, 2020). It presents countries with major political and public health challenges. On 20 January 2020, the national health and Health Commission of China included COVID‐19 in Class B infectious diseases and border health quarantine infectious diseases and launched the first‐level response to major public health emergencies in many provinces according to class A infectious disease management (Commission, 2020). Currently, more than 380 medical teams and more than 42,000 medical personnel have come to Hubei to fight against COVID‐19. In 26 January, *Novel Coronavirus Infection Prevention and Control of Common Medical Protective* articles were issued by the National Health Protection Commission (Trial). It pointed out that medical personnel should use medical masks, goggles, protective mask/face screen, isolation clothing, protective clothing and latex inspection hand in the front‐line areas of fever clinic, isolation ward and isolation intensive care unit (area) (Jie Xia, Cao, Zhang, Chan, & Wang, 2020).

Novel coronavirus front‐line medical staff are facing high risk of exposure (Zhou, Huang, Xiao, Huang, & Fan, 2020). They need to wear medical protective equipment for a long time in the crowded and moist environment of protective clothing. It is very easy to cause local persistent pressure ischaemia and the skin problems such as nasal and facial pressure injuries (Kayser, VanGilder, Ayello, & Lachenbruch, 2018; Wen Huang, Wang, Xiao, & Li, 2020). As an important component of personal socialization, appearance abnormality caused by skin injury of nose and face is easy to trigger individual's dissatisfaction with their appearance and cause social appearance anxiety (van den Elzen et al., 2012), even affect work enthusiasm and enthusiasm. Research shows that (Davis, Dionne, & Shuster, 2001; Zhang, Chen, Wang, Xie, & Qiu, 2015) personality traits and appearance tendency have a certain linear correlation. Neuroticism personality attaches great importance to personal appearance, and it is easier to produce appearance anxiety (Martin & Racine, 2017). There is a correlation between personality traits and nurses' emotions, which can lead to the difference of risk decision‐making by affecting their emotions (Zhang, 2012).

### 1.1 Research question

This study describes the COVID‐19 epidemic situation and the psychological state of medical staff who had the nasal and facial pressure ulcers caused by the use of protective devices during the outbreak of new coronavirus and explores the correlation between different personality traits, positive and negative emotions and social appearance anxiety, in order to alleviate the degree of anxiety of the medical staff exposed to the nasal and facial pressure ulcers and provide more individual psychological support.

## 2 METHODS

### 2.1 Design and sample size

This was a cross‐sectional study, which collected data using convenience sampling, between February and March 2020. Potential participants were those medical teams affiliated three hospitals of a university in Hunan Province sent to Hubei Province for epidemic prevention. A total of 210 questionnaires were sent out, and 207 effective questionnaires were finally recovered after screening. The effective rate of the questionnaires was 98.6%. Inclusion criterion was volunteered to participate in the study. Exclusion criterion was incomplete content. All the questionnaires were in the form of anonymity and confidentiality.

### 2.2 Measurement of variables

#### 2.2.1 Demographic information

Gender identity, age, BMI, accumulated first‐line working time, average working hours per shift, preventive application of pressure sore, discomfort of wearing protective equipment, presence of symptoms related to pressure ulcers and other.

#### 2.2.2 Eysenck Personality Questionnaire Short Scale (EPQ‐RSC)

The scale was compiled by Ming‐yi Qian, Zhu, and Zhang (2000) and consists of four subscales: E (extraversion), N (neuroticism), P (psychoticism) and L (lie). They were used to measure the subjects' internal and external disposition, emotional stability, mental deviation and lying tendency. Cronbach's α coefficient of this scale is 0.79–0.84.

#### 2.2.3 Social Appearance Anxiety Scale (SAAS)

The scale was compiled by Hart et al. (2008) based on social anxiety level, self‐image dissatisfaction and body deformation disorder measurement standard, translated by Kong Shanshan and Yang Hongfei, to evaluate the anxiety sensitivity related to appearance and the overall face of the individual (Shan‐shan Kong, 2009). There are 16 items in this scale. The higher the score, the more serious the anxiety about social appearance. In this study, the internal consistency coefficient is 0.9288.

#### 2.2.4 Positive and Negative Affect Scale (PANAS)

There are 20 words describing emotion in the scale, which can be divided into two subscales: positive and negative emotion (Huang, Yang, & Li, 2003). A scale of 1 (few) to 5 (very frequent) was used. A high positive emotional score indicates an individual's active and engaged emotional state; a high negative emotional score indicates an individual's low mood. In this study, Cronbach's *α* coefficients of these scales were 0.85 and 0.83, respectively.

### 2.3 Analytic strategy

The questionnaire was collected, and the data were sorted out. SPSS 25.0 software was used to build the database for analysis. Take *α* = 0.05 as the test level, and *p* value takes the bilateral probability. The measurement data were described by mean ± standard deviation, the counting data were described by the number or percentage of cases, the influencing factors of stress injury events were analysed by chi‐square test and logistic regression, the differences of social appearance anxiety scores were compared by the method of propensity score matching and *t* test of two samples, and the correlation between three scales was calculated by Pearson correlation analysis. The mediation effect was assessed by PROCESS module 4 in SPSS. Test criteria for mediation effect were as follows: ① the independent variable must be related to the intermediate variable; ② the independent variable must be related to the dependent variable; ③ when controlling the intermediate variable, the correlation between the independent variable and the dependent variable decreases significantly, if it is still statistically significant after the decline, it is the site mediating effect, if it becomes not statistically significant after the decline, it is the complete mediating effect (Zhong‐lin Wen, Hou, & Liu, 2004).

### 2.4 Ethic issues

After obtaining permission from the Hospital Ethics Committee, the researchers distributed questionnaires to medical staff who met the inclusion criteria and participated voluntarily, with informing the purpose of the study.

## 3 RESULTS

### 3.1 Characteristics

A total of 207 first‐line medical staff ranged from 19–49 years of age were surveyed, including 46 males (22.2%) and 161 women (77.8%). The majority were nurses (81.2%), and the others were doctors. More than half of them (50.7%) working in first line for 15–30 days and 36 people (17.4%) in 30–60 days, with 45 (21.7%) less than 15 days. The mainly average duration per shift was 4–6 hr, with 25.6% were 7–8 hr. During the epidemic period, the protective masks worn by the medical staff were all head‐worn. 81.6% of the respondents felt discomfort such as nasal tenderness (88.7%) and ear pain (89.3%) after wearing the protective equipment for 2–4 hr (41.1%), and the pain score was (5.13 ± 2.77). 46.9% of the medical staff said that they did not use the preventive dressing of pressure ulcers during work.

### 3.2 Risk factors of nasal and facial MDR PUs

The results showed that 192 (92.8%) of the respondents had pressure ulcers caused by using protective equipment, and the symptoms were erythema (91.6%) with intact local skin and invariable white pressure, or with complete serous blister (8.4%). About 79.2% of the medical staff reported that these symptoms could be relieved by themselves within 2 days, and 1.4% could not be relieved by themselves unless other measures were needed. ANOVA showed that there was a statistically significant difference between the age and the degree of pain in wearing protective equipment (*p* < .05) (Table 1).

**Table 1:** ANOVA on nasal and facial MDR PUs of anti‐epidemic medical staff (total = 207)

Variables		Have MDR PUs	Not MDR PUs	*χ* ^2^	*p*
Sexual	Male	40	6	2.96	.085
	Female	152	9		
Age groups	Under 20	0	1	13.22	.004
	20–29	119	9		
	30–39	65	4		
	40–49	8	1		
BMI groups	Underweight	19	2	1.1	.778
	Normal weight	157	11		
	Overweight	14	2		
	Obese	2	0		
Frequency of physical exercise (every month)	Never	74	5	3.63	.459
	＜5 times	66	4		
	5–10 times	30	5		
	10–15 times	13	1		
	＞15 times	9	0		
Total anti‐epidemic days	<15	41	4	5.65	.13
	15–30	100	5		
	30–60	34	2		
	More than 60	17	4		
Average hours per day	<4	10	2	2.11	.55
	4–6	101	6		
	7–8	49	4		
	More than 8	32	3		
Frequency of preventive dressing (every week)	Never	87	10	3.95	.267
	1–2 times	42	3		
	3–4 times	32	0		
	Always	31	2		
Whether there is discomfort during wearing protective gear	Yes	159	10	2.42	.12
	No	33	5		
Pain assessment during wearing protective gear	No pain	25	4	11.2	.011
	Mild pain	21	5		
	Moderate pain	75	5		
	Severe pain	71	1		

The variables with statistical significance in single factor analysis are included in the regression model, and the binary logistic regression model is established with whether there are pressure ulcers as the dependent variable and age and pain degree as the independent variable (Table 2). The analysis shows that the less conscious pain of medical staff in using protective equipment, the greater the possibility of pressure ulcers.

**Table 2:** Logistic regression on nasal and facial MDR PUs of anti‐epidemic medical staff

Variable	*B*	*SE*	Wald *χ* ^2^	*p*	Exp (*B*)	95.0% CI for Exp (*B*)	
						Lower	Upper
The degree of pain	−0.700	0.252	7.731	.005	0.497	0.303	0.813
Age	−0.486	0.488	0.993	.319	0.615	0.236	1.600
constant	−0.721	0.636	1.285	.257	0.486		

### 3.3 Personality traits, positive and negative emotions and social appearance anxiety of anti‐epidemic medical staff

The scores of extraversion, neuroticism, psychoticism and lie were (50.58 ± 10.98), (51.16 ± 11.71), (50.30 ± 8.46) and (55.00 ± 8.68), respectively; the scores of positive and negative emotions were (33.0 ± 6.30) and (24.60 ± 7.37), respectively, during the anti‐epidemic period; and the scores of social appearance anxiety of medical staff with and without MDR PUs were (40.72 ± 14.48), (36.73 ± 12.93) and (*t* = 2.548, *p* = .017). There was a statistical difference in the scores of social appearance anxiety between the medical staff with and without nasal and facial MDR PUs.

### 3.4 Correlation analysis between personality traits, positive and negative emotions and social anxiety

The correlations among personality traits, social appearance anxiety, positive and negative emotions are presented in Table 3. Extraversion has a statistically significant positive correlation with positive emotions during the epidemic (*r* = 0.180, *p *< .05), a statistically significant negative correlation with negative emotions (*r* = −0.147, *p *< .05) and no statistically significant correlation with appearance anxiety; negative emotions and appearance anxiety related to nasal and facial pressure ulcers were positively correlated with neuroticism (*r* = 0.245, *r* = 0.330, respectively, *p *< .05). In addition, positive emotions during the outbreak were significantly negatively correlated with neuroticism (*r *= −0.260, *p *< .05). Also, lie was significantly positively correlated with negative emotions during the COVID‐19 epidemic (*r* = 0.138, *p *< .05).

**Table 3:** Correlations between personality traits, positive and negative emotions and MDR PUs‐related SAAS scores in medical staff (*r*)

Items	Extraversion	Neuroticism	Lie	Psychoticism	SAAS	Positive
Neuroticism	−0.089					
Lie	−0.170**	−0.021				
Psychoticism	−0.133	−0.024	0.089**			
SAAS^a^	−0.060	0.330**	0.107	0.095		
Positive^b^	0.180**	−0.260**	−0.092	−0.008	−0.028	
Negative^c^	−0.147**	0.245**	0.138**	0.111	0.430**	−0.231**

^a^ SAAS means Social Appearance Anxiety Scale.

^b^ Positive Emotion Scale.

^c^ Negative Emotion Scale.

** Statistically significant at *p* < .05.

### 3.5 Negative emotion as a mediator variable in the association between neuroticism and SAAS

**Figure 1:**
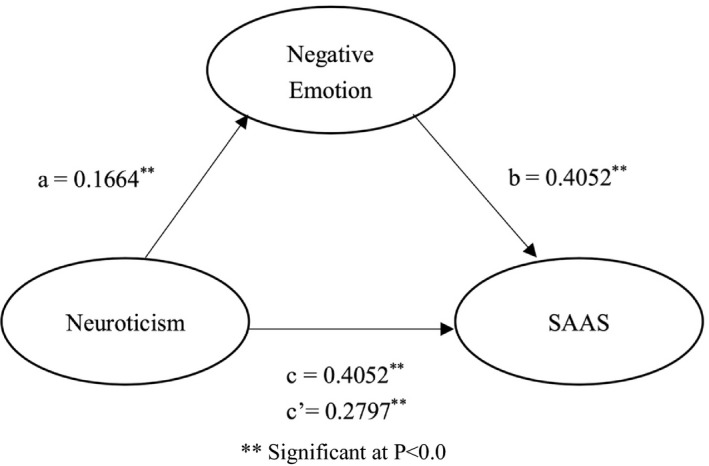
The intermediary role of negative emotion in the neuroticism and SAAS (Social Appearance Anxiety Scale). ^**^Statistically significant at *p* < .01

We further investigated the mediating role of negative emotion in the relationship between neuroticism and social appearance anxiety (SAAS). The mediation model was set up with neuroticism personality as independent variable and SAAS as dependent variable under the control of gender, age and occupation. The negative emotion score was inserted in model as a mediator. The results are shown in Tables 4 and 5 and Figure 1.

**Table 4:** The mediating effect of Negative Emotion

Outcome variable	Prediction variable	*R*	*R* ^2^	*F*	*β*	*t*	*p*
SAAS^a^		0.3354	0.1125	6.3708			
	Sexual				2.0897	0.7852	.4333
	Age				−0.2987	−0.1734	.8625
	Occupation				0.4230	0.1520	.8793
	Neuroticism				0.4052	4.8798	<.0001
Negative^b^		0.2886	0.0833	4.5645			
	Sexual				−2.5801	−1.8657	.0635
	Age				0.7278	0.8129	.4172
	Occupation				2.8769	1.9896	.4172
	Neuroticism				0.1664	3.8576	.0002
SAAS		0.4987	0.2487	13.241			
	Sexual				4.0347	1.6296	.1048
	Age				−0.8475	−0.5324	.5951
	Occupation				−1.7459	−0.6736	.5013
	Neuroticism				0.2797	3.5240	.0005
	Negative				0.7539	0.6862	<.0001

^a^ SAAS means Social Appearance Anxiety Scale.

^b^ Negative means negative emotion of medical staff.

**Table 5:** Breakdown of total effect, direct effect and intermediate effect

	Effect	*P*	Boot *SE*	Boot LL CI	Boot UL CI	Relative effect
Total effect	0.4052	<.01	0.0830	0.2415	0.5690	
Direct effect	0.2797	<.01	0.0794	0.1232	0.4363	69.03%
Indirect effect	0.1255	<.01	0.0381	0.0546	0.2050	30.97%

As can be seen, the mediation analysis confirmed a statistically significant positive effect of neuroticism on negative emotion, which proved to be a positive predictor of SAAS. A statistically significant total effect was also found of neuroticism on SAAS (*β* = 0.4052, *p* < .01, Boot *SE* = 0.0830, 95% CI = 0.2415–0.5690); however, this effect lessened when the mediator was entered into the model, which suggests a partial mediating effect. The bootstrap procedure was statistically significant for the indirect of neuroticism through negative emotion (*β* = 0.1255, Boot *SE* = 0.0381, 95% CI = 0.0546–0.2050). These results illustrate the important of the mediating role of negative emotion as strong predictor of SAAS, and the mediation effect ratio was 30.97%.

## 4 DISCUSSION

### 4.1 The current situation of pressure ulcers of nose and face of medical staff during the outbreak of novel coronavirus

The situation became extremely severe and complicated due to the global spread of the virus. Medical staff have been fighting in the clinical front line. In order to resist the invasion and infection of the virus, it is necessary to wear all kinds of medical protective equipment for a long time to ensure their own safety and effectively block the transmission of the virus among individuals. According to the existing data (Wen Huang et al., 2020), medical device‐related pressure ulcers (MDR PUs) can be easily induced by the superposition of respirators, goggles and other protective devices. In addition to the huge risk of infection, there are more and more pressure injuries on nose and face caused by wearing protective equipment, which seriously threaten the physical and mental health of medical staff.

In 2010, Black et al. (2010) first proposed the concept of MDR PUs and pointed out that the incidence of MDR PUs was 0.9%–41.2%. In this study, 92.8% of the anti‐epidemic medical staff suffered pressure ulcers on the nose and face, and the symptoms were mainly manifested as intact local skin, constant white erythema or complete serous blisters (Wan‐li Zhu, 2018). Due to the protective equipment used by medical staff, such as hard goggles and N95 nose metal fixed piece, the pressure and friction of local tissues are increased (Jaul, 2011), and wearing protective equipment for a long time prolongs the pressure time of the same part of the skin, and the poor permeability of protective clothing increases the moisture content of the skin, all of the above factors increase the risk of MDR PUs of medical staff.

It is reported that the parts of MDR PUs of medical staff during the epidemic are common in the nose, cheek, forehead and the back of auricle (Wen Huang et al., 2020). Because of the thin muscular layer, lack of fat tissue and superficial position of the bridge of nose and the back of auricle, it is more likely to produce pressure ulcers and other skin problems. In our study, the medical staff who wear protective equipment have the discomfort of nasal tenderness and pain in the back of auricle, which is consistent with the previous literature.

Further logistic regression analysis shows that the higher the degree of pain in wearing protective equipment, the less the possibility of pressure ulcers. Analysis of the reasons may be that when medical staff wear N95 masks or other protective equipment, if they feel strongly uncomfortable, they will try to adjust the tightness or change the equipment, effectively alleviating the state of continuous pressure on the same part, and continuous pressure is the key risk factor for the formation of pressure ulcers (Qiu‐xia Huang, Tang, Wang, Zhou, & Zhan, 2018). At present, for the high‐risk groups such as anti‐epidemic medical staff, it is recommended to use liquid dressing with hydrocolloid dressing for local skincare in the prone areas of pressure ulcers such as nose, face and ear and properly keep the face clean and moist, which can effectively reduce the incidence of pressure ulcers on the nose and face of nursing staff and alleviate the severity of the ulcers (Jie Xia et al., 2020). If the protective equipment can be improved to make it more fit to the face and increase comfort (Li et al., 2020), the production of MDR PUs can be further reduced.

### 4.2 Mental state of medical staff with pressure ulcers of nose and face

As the carrier of human body image and appearance, appearance is not only the symbol of individual as social natural person, but also the function of transmitting social information and performing social functions (Yaman, 2017). It is one of the characteristics that human beings are most vulnerable to the influence of external factors (Frevert & Walker, 2014). The gap between human physical appearance and ideal appearance is an important cause of social appearance anxiety.

The age range of medical staff investigated in this study was between 20–40 years old. With the rapid improvement of self‐awareness, both men and women in this age group pay more attention to their appearance. Appearance, as one of the important factors of personal socialization, the skin problem of pressure ulcers on nose and face widens the gap between medical staff and their ideal appearance, increases the anxiety level of fear of others' evaluation of themselves and easily triggers individual's dissatisfaction with their appearance and causes social appearance anxiety (van den Elzen et al., 2012). In this study, the scores of social appearance anxiety of medical staff with and without stress injury were (40.72 ± 14.48) (36.73 ± 12.93), respectively (*t* = 2.548, *p* = .017), which indicated that the occurrence of stress injury increased their anxiety about their own appearance. However, the social appearance anxiety of human beings will have a negative impact on their lives (Yaman, 2017). This psychological feature may affect the work mood of anti‐epidemic medical staff, leading to a decline in their work enthusiasm during the epidemic (He et al., 2012).

### 4.3 Analysis of the relationship between personality traits, appearance anxiety, positive and negative emotional variables

Personality is a relatively stable psychological trait formed by individuals in social life and an important factor affecting mental health (Hopwood et al., 2008). In this study, neuroticism was positively correlated with the appearance anxiety related to the stress injury of nose and face and the negative emotion during the epidemic period. That is to say, the neuroticism of medical staff tended to be unstable after the stress injury of nose and face showed more anxiety and worry about their external image and was more likely to produce negative emotions such as panic and depression at work. Research (Bin zhang, 2017; Davis et al., 2001) also confirmed that neuroticism is related to appearance orientation. High scores on neuroticism factors show that individuals pay more attention to appearance, and individuals with neuroticism personality have the characteristics of excitability, emotionalization, anxiety, etc., and tend to adopt negative ways to deal with high‐pressure environment, such as escape, self‐blame, etc., which is a powerful predictor of negative emotions.

Extraversion personality trait was positively correlated with positive emotion during the COVID‐19 pandemic and negatively correlated with negative emotions. Extraversion personality of medical staff showed less anxiety and depression during the fight against novel coronavirus and tended to deal with difficulties and challenges positively. This is similar to the research results of Zhang et al. (2015), etc., when the extroverted personality individuals encounter stress events, the self‐efficacy of emotion regulation is strong, which is conducive to resolving negative emotions (Ping Zhang & Wang, 2015). In addition, in the positive emotional state, the extroverted personality individuals will show stronger creativity, higher efficiency of problem‐solving and more comprehensive decision‐making (Zhang, 2012). Therefore, targeted psychological intervention and support for different personality traits of anti‐epidemic medical staff will be conducive to the cultivation of good psychological status of medical staff and improve the quality of medical care.

### 4.4 Intermediary effect of negative emotion during COVID‐19

This study revealed that negative emotion could be a mediator between neuroticism and SAAS and the mediation analyses are consistent with previous studies that have demonstrated that neuroticism to be a predictor of negative emotion, and the negative emotion to be a predictor of social appearance anxiety (Mason et al., 2018; Noteboom, Beekman, Vogelzangs, & Penninx, 2016; Zhang & Zheng, 2019). The heavy workload for fighting the novel coronavirus could directly cause negative emotions of medical staff and high risk of infection. If the individual of neuroticism trait is in a state of negative emotion, they would be overly concerned about their external appearance. If long‐term high‐intensity work has been completed, the symptoms including pressure and blisters on the nasal surface cannot be dissipated, and the anxiety degree of the physiognomy will be strengthened. This finding points to the importance of specific preventive measures tailored to medical staff of neuroticism trait with high levels of appearance anxiety to alleviate negative emotion. In order to reduce the negative emotions of medical staff in the front line of anti‐epidemic, we should strengthen social support and improve the level of enthusiasm and emotion of them on the basis of the improvement measures such as the application of preventive dressing and the matching of medical protective equipment with appropriate tightness. Therefore, many social support measures have been put forward in China, such as improving the salary, arranging the rotation of high‐intensity medical workers in the front line, carrying out active health monitoring and setting up psychological support hotlines.

Good personality characteristics of medical staff include noble moral sense and sincere sympathy, positive and stable emotions, good interpersonal relationship and communication ability (Li, Li, & Li, 2006). In order to cultivate good personality characteristics and improve psychological quality of medical staff, efforts should be made in the following aspects: special personnel should regularly carry out emotional management of anti‐epidemic workers, pay attention to the construction of psychological quality of medical staff, carry out psychological intervention and counselling focusing on front‐line medical staff, strengthen the propaganda of professional ethics, strengthen the sense of responsibility of medical staff, carry out appropriate development of doctors and patients during the epidemic, interactive activities between doctors and nurses, enhance the environmental construction of humanistic care and improve interpersonal relationship and communication ability. In summary, neuroticism trait has both direct and indirect effects on social appearance anxiety through negative emotion. It would support the idea that preventive measures should tackle emotional aspects by providing strategies for managing and regulating negative emotions.

The findings of this study have to be seen in the light of some limitations included the following: (a) all the data were implementation by self‐report questionnaires online, which means that they can be influenced by participants' subjective responses; and (b) the small sample size may not be representative of the Chinese front‐line medical staff population, and therefore, the findings are not necessarily generalizable to other medical staff on the same period. It is suggested to conduct further studies due to the current global pandemic situation and limitations of this regard. Negative emotion partially mediated the relationship between neuroticism trait and social appearance anxiety was identified in a selected population of first‐line medical staff, and it was a strength of research. Hence, it is suggested that government should consider personality dimensions and negative emotions of medical staff who fight against COVID‐19 that promote medical staff's mental health.

## 5 CONCLUSIONS

Medical staff treating the COVID‐19 pandemic are easily suffered the nasal and facial pressure ulcers. During the epidemic period, the neuroticism trait of medical staff can not only directly affect their social appearance anxiety, but also indirectly affect them through negative emotion as intermediary variables. Therefore, while actively developing comfortable protective equipment and adopting appropriate preventive dressings to protect the nose and face skin of medical staff, we should also pay more attention to improve their psychological quality and emotional self‐management ability and relieve their anxiety about the appearance of the nose and face stress injury, so as to defeat the COVID‐19 safely.

## CONFLICT OF INTEREST

The authors have no conflicts of interest relevant to this article.

## AUTHOR CONTRIBUTIONS

Xiaodi Kong, Yong Cao, Xiaonian Luo and Lianxiang He conceptualized, planned and implemented the study. Xiaodi Kong completed the analysis with input from Xiaonian Luo. Xiaodi Kong and Yong Cao drafted the article and all authors critically examined, revised and approved the final version.

## PATIENT CONSENT STATEMENT

There was oral informed consent between medical staff.
